# Multiomics analysis to evaluate the enrichment of extracellular vesicles from human plasma

**DOI:** 10.1016/j.jlr.2025.100877

**Published:** 2025-08-19

**Authors:** Huaqi Su, Christopher Fowler, Colin L. Masters, Kevin J. Barnham, Gavin E. Reid, Laura J. Vella

**Affiliations:** 1 The Florey Institute of Neuroscience and Mental Health, Melbourne, Australia; 2 Florey Department of Neuroscience and Mental Health, The University of Melbourne, Parkville, Victoria, Australia; 3 School of Chemistry, The University of Melbourne, Parkville, Victoria, Australia; 4 Department of Biochemistry and Pharmacology, The University of Melbourne, Parkville, Victoria, Australia; 5 Bio21 Molecular Science and Biotechnology Institute, The University of Melbourne, Parkville, Victoria, Australia

**Keywords:** blood, plasma, extracellular vesicles, exosomes, lipidomics, proteomics, proteins, cholesteryl esters

## Abstract

Extracellular vesicles (EVs) in blood plasma offer a valuable reservoir of intracellular cellular cargo, making them a promising source of liquid-based biomarkers. However, the complexity of plasma, with its abundance of non-EV particles and plasma proteins, presents challenges for their molecular characterization, particular their lipid composition, using mass spectrometry–based technologies. Consequently, there is currently no comprehensive blueprint detailing both the proteomes and lipidomes of highly enriched plasma EVs. We employed an orthogonal approach using density gradient ultracentrifugation (DGUC) and size-exclusion chromatography (SEC) to isolate EVs and conducted a comparative study on four different SEC columns following DGUC to evaluate the capacity of the SEC columns in enriching EVs while depleting plasma proteins and lipoprotein particles. The EV fractions were analyzed with data-independent acquisition proteomics and nano-ESI-ultrahigh-resolution accurate mass spectrometric lipidomics. DGUC followed by the appropriated sized SEC provided the best enrichment of EVs and the corresponding depletion of plasma protein and lipoprotein particle contaminants. We show that glycerophosphoethanoamine, glycerophosphoserine, ceramide, and sphingomyelin lipids are significantly enriched, while cholesteryl ester content is significantly depleted in EVs compared to platelet depleted plasma. This strategy also enabled the detection of proteins in the enriched EV fractions with functions related to mitochondria, endosomal-autophagic-lysosomal pathways, and the central nervous system. This study highlights the benefit of depleting coisolates from plasma EV preparations to enable the detection of proteins and lipids with potential future clinical utility and underscores the need for ongoing development of improved high-throughput EV isolation technologies.

Extracellular vesicles (EVs) are lipid membrane vesicles that are released by all cell types. They are heterogeneous and can be classified into various categories based on their size, cellular origin and biogenesis pathways. EVs are present in bio-fluids such as blood, urine and breast milk (1, 2) and carry cargo including nucleic acids, proteins, and lipids that can report on the pathophysiological condition of the parent cell.

In recent years, there has been increasing interest in examining EVs in blood, with the aim of using EVs as a source of biomarkers for disease diagnosis, prognosis, and therapeutic monitoring (1, 3, 4, 5, 6, 7). However, the complexity of blood and the challenges in effectively separating EVs from more abundant blood components (e.g., albumin and lipoprotein particles) have made it difficult to clearly define the lipidomic profile of EVs in blood under normal physiological conditions (8). Chylomicrons and LDL particles overlap in size with EVs, while EVs share a similar density with HDL particles (8, 9). Since lipoprotein particles are lipid-rich, their copurification can severely impact biomolecular analyses, with diminished coverage of EV lipids and the generation of potentially misleading data (10, 11, 12).

Several studies have endeavored to analyze the proteome and lipidome of EVs isolated from plasma and serum, however, the findings are limited by shortcomings in the isolation methodologies employed. Some studies used size-exclusion chromatography (SEC) (13, 14, 15) or density gradient alone (16), which can coisolate lipoprotein particles of similar size or density to EVs, respectively. Others have employed methods such as sequential ultracentrifugation (16, 17), polymer precipitation (18, 19), or organic solvent precipitation (20, 21), which coisolate and or precipitate lipoproteins and or abundant plasma proteins. While there are three studies that have used more robust techniques (SEC combined with density gradient ultracentrifugation (DGUC) or phosphatidylserine affinity-based methods) to isolate plasma EVs, none have examined the “enriched” EV lipidome compared to platelet depleted plasma (PDP) and HDL (22, 23, 24).

Here, we evaluated and optimized an orthogonal method (22), employing DGUC and SEC to isolate EVs. As a measure of EV enrichment EV fractions were subjected to data-independent acquisition (DIA)-LC-MS/MS proteomics and direct infusion nano-ESI-ultrahigh resolution accurate MS lipidomics. This approach enabled enhanced detection of low abundant EV proteins associated with mitochondria and endosomal-autophagic-lysosomal (EAL) pathways and the central nervous system (CNS) and allowed evaluation of the level of lipid enrichment/depletion in EVs compared to PDP and high-density (HD) fractions from DGUC.

While this study requires future validation with a larger and more diverse cohort, the remarkably consistent lipid profile, that is, enrichment of sphingolipids (SPs) and depletion of cholesteryl ester (CE) lipids observed across EVs relative to PDP and HD samples suggests that the EV lipid signature in plasma may be highly conserved. This multiomics dataset provides the research community with further evidence highlighting the importance of depleting blood contaminants, specifically lipoprotein particles, during isolation of EVs from plasma. The dataset further suggests that plasma EVs could offer insight into intracellular networks, thereby aiding future investigations into the potential of blood EV biomarkers for disease diagnosis and monitoring.

## Materials and methods

### Blood sample collection, processing, and ethics

Peripheral blood was collected with informed consent from healthy adult donors after overnight fasting. Blood was drawn through a Safety-Lok, 21G needle collection set (BD Vacutainer) and collected in K2E EDTA tubes (BD Vacutainer). Blood was visually inspected for hemolysis, and no hemolyzed samples were used. The whole blood collected in K2E EDTA lavender top BD vacutainer was inverted 10 times to mix with EDTA and stored upright at ambient temperature until plasma processing. Within 2 h, blood was processed to separate PDP for EV isolation. The first tube of collected blood was not discarded but retained for plasma processing. Samples were centrifuged at 1,500 *g* at 20°C for 20 min. Plasma was aspirated from 1 cm above the buffy coat and transferred to 15 ml screw cap tubes. Plasma was centrifuged at 2,500 *g* at 20°C for 10 min, twice, to deplete platelets. The PDP was then aliquoted and stored at −80°C until use. The MIBlood EV check list for Standardized Reporting Tool for Blood EV Research (Human) is provided as a supplementary file. Blood handling and experimental procedures were approved by The University of Melbourne human ethics committee (ethics application number: 24582) and in accordance with the National Health and Medical Research Council guidelines. The study protocol adhered to the ethical principles outlined in the Word Medical Association’s Declaration of Helsinki.

### Plasma EVs isolation via OptiPrep™ DGUC and SEC

**Fig. 1:**
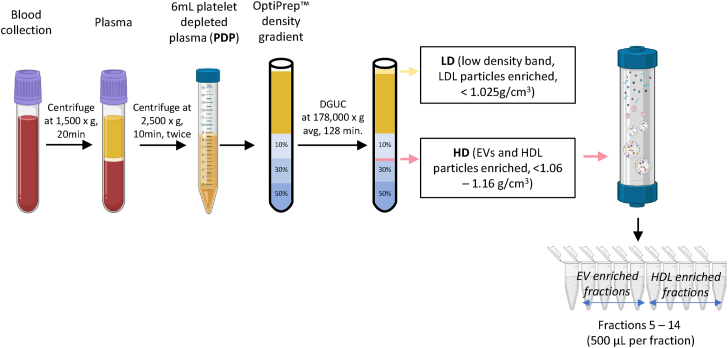
Schematic overview of the plasma EVs isolation workflow. Blood samples were collected in K2E EDTA tubes and spun at 1,500 *g* for 20 min to separate plasma. Plasma was spun at 2,500 *g* for 10 min twice to deplete platelets from the plasma. Platelet depleted plasma (PDP) was collected and stored at −80°C until use. For EVs isolation, 6 ml of PDP was layered on top of an OptiPrep™ cushion and subjected to 178,000 *g* avg, 4°C, for 128 min in a SW40 rotor. Aliquots of LD and HD bands were collected for downstream immunoblotting and omics analyses. The remaining HD fractions were pooled before SEC column separation (Sepharose® CL-6B (24 nm), IZON qEVoriginal™ Legacy 35 nm, Sepharose® CL-4B (42 nm), and IZON qEVoriginal™ Legacy 70 nm). EV = extracellular vesicles, LD = low density, HD = high density.

Plasma EV isolation followed the previously published protocol from Karimi *et al.* (22) with modifications. For SEC column comparison, a pooled PDP sample was used. A schematic of the workflow is shown in Figure 1. Frozen PDP was thawed at 37°C. Before proceeding to plasma EV isolation, a 2 μl aliquot of pooled PDP was retained for protein quantitation and immunoblotting and a 20 μl aliquot was retained for “omic” characterization.

An OptiPrep™ density cushion was prepared whereby 2 ml of 50% OptiPrep™ (Sigma-Aldrich) PBS (Thermo Fisher Scientific) solution was layered at the bottom of SW40Ti ultraclear tube (Beckman Coulter), followed by 2 ml of 30% OptiPrep™-PBS solution, and 2 ml of 10% OptiPrep™-PBS solution. Approximately 6.5 ml of PDP was loaded on top of each OptiPrep™ density gradient and a total of 16 density gradients were used. The OptiPrep™ density gradients were subjected to ultracentrifugation at 178,000 *g* avg, at 4°C for 128 min using a SW 40Ti rotor (Beckman Coulter). After centrifugation, a visible band floating on top of the gradient, containing LDL particles, was collected, and annotated as low-density (LD) band, and a visible band between the 10% and 30% OptiPrep™ layers, containing EVs and HDL particles, was collected and annotated as HD band. All LD and HD bands collected were pooled, respectively, and a 10 μl aliquot from LD band and a 20 μl aliquot from HD band were retained for protein quantitation and immunoblotting and a 65 μl aliquot from HD band was retained for “omic” characterization. The pooled HD samples were made up to 8 ml with PBS and equally divided into 16 aliquots of 500 μl (4 aliquots for each of the 4 SEC column types) and loaded onto IZON qEVoriginal™ 35 nm or 70 nm (IZON Science), or home-made Sepharose™ CL-4B or CL-6B (Sigma Aldrich) SEC columns. The home-made Sepharose™ CL-4B and CL-6B SEC columns were packed following a published protocol (25). Briefly, Sepharose™ CL-4B and CL-6B resins (Sigma-Aldrich) were washed with 0.32% citrate in PBS and packed in a 10 ml disposable syringe (Hapool Medical Technology) to a final volume of 10 ml and equilibrated with PBS before use. Separation of content in HD with each SEC column type was repeated four times. SEC performance was carried out at room temperature, and all columns were equilibrated with 30 ml of PBS solution before loading HD. For all SEC fraction collection, fractions of 500 μl were collected manually, the first 2 ml elution (fractions F1–F4) was discarded and fractions F5–F14 were collected for characterization. All columns were washed and equilibrated after elution and before the next aliquot of the HD was loaded. Reproducibility of SEC performance was determined in preliminary experiments (data not shown) and all SEC columns (both home-made and those from IZON) were used up to 4 times as recommended by the supplier.

Fractions from multiple SEC columns were pooled (fractions F5–F14, a total of 2 ml each) followed by addition of 105.3 μl of 20X PhosSTOP phosphatase inhibitor (Sigma-Aldrich)/cOmplete protease inhibitor (including EDTA, Sigma-Aldrich) PBS solution. For protein quantitation and immunoblotting, an aliquot of 500 μl from each fraction was mixed with RIPA buffer and sonicated in ice-cold water bath for 20 min. Protein precipitation was performed by incubating in 20% trichloroacetic acid on ice for 30 min followed by centrifugation at 16,000 *g* for 20 min at 4°C and pellet was washed with 500 μl of −20°C 100% acetone and spun at 16,000 *g* for 15 min at 4°C, twice, to remove trichloroacetic acid. Protein pellets were air-dried overnight and resuspended in 30 μl PBS containing 2% SDS, heated at 95°C for 10 min, and proceeded to 20 min water bath sonication before protein quantitation. The remaining combined SEC fractions were freeze-dried and proceeded to lipid and protein extraction.

### SDS-PAGE and immunoblotting

PDP, LD, and HD samples were mixed with phosphatase inhibitor and protease inhibitor PBS solution and RIPA buffer and sonicated in ice-cold water bath for 20 min. Protein content was determined using bovine serum albumin as standard with the Pierce™ BCA assay kit (catalog number #23225, Thermo Fisher Scientific) according to the manufacturer’s instructions. PDP, LD, HD samples, and equal volume of all SEC protein fractions were prepared in 4X NuPAGE™ LDS sample buffer and 10X Bolt™ sample reducing agent and boiled (10 min, 90°C) followed by centrifugation (16,000 *g*, 1 min). Normalized PDP, LD, and HD samples (2.3 μg) and equal volume for all SEC fractions were electrophoresed on 4–12% NuPAGE™ Bis-Tris in NuPAGE™ MES SDS running buffer (Thermo Fisher Scientific) for 60 min at 160 V. Proteins were transferred onto nitrocellulose membranes using an iBlot™ 2 Dry Blotting System (Thermo Fisher Scientific). Total protein profile on the membranes was stained using Revert™ 700 Total Protein Stain following instruction (LI-COR) and imaged on an Odyssey® Fc Imaging System (LI-COR).

After removing staining, membranes were blocked with 1X Blocker™ FL Fluorescent Blocking Buffer (Thermo Fisher Scientific) for 1 h at room temperature, followed by 48 h incubation with primary antibodies (syntenin #ab133267 from Abcam, flotillin-1 #610821 from BD Biosciences; ApoA-1 #3350 from Cell Signaling Technology and ApoB #139401 from Abcam) at 4°C. Membranes were washed 4 times with tris-buffered saline with Tween 20 (TBS-T, 30 min, room temperature) following a 1 h, room temperature incubation of the IRDye® 800CW Goat anti-Rabbit IgG secondary antibody (#925-32211, LICOR) or the IRDye® 800CW Goat anti-Mouse IgG secondary antibody (#926-32210, LICOR). All antibodies were diluted in TBS-T. Membranes were washed 4 times with TBS-T (30 min, room temperature). The membranes were visualized on an Odyssey® Fc Imaging System (LI-COR).

### Lipid extraction from plasma and derived fractions

Freeze-dried SEC fraction samples, along with PDP (20 μl) and HD (65 μl) samples, were prepared in 200 μl ice-cold 60% methanol (LC-MS grade, EMD Millipore Corporation) containing 0.01% (w/v) butylated hydroxytoluene (BHT, Sigma-Aldrich) in Safe-Lock Eppendorf centrifuge tubes. All samples were sonicated in an ice-cold water bath sonicator for 20 min prior to monophasic lipid extraction following a method previously reported (26, 27). Briefly, 120 μl of MilliQ water, 420 μl of methanol with 0.01% (w/v) BHT, and a total of 270 μl of chloroform with 0.01% (w/v) BHT (containing internal lipid standards, Supplemental Table S1) were added to all samples, followed by 10 s vortexing and agitation at 1,000 rpm for 30 min at room temperature. Samples were spun at 14,000 rpm for 15 min at room temperature and supernatants containing lipids were transferred to new tubes. One hundred milliliters of MilliQ water and 400 μl of chloroform:methanol (1:2, v:v) containing 0.01% (w/v) BHT (BHT) were added to remaining pellets for re-extraction to maximise lipid recovery following incubation and centrifugation as described above. Lipid extract supernatants from the same extractions were pooled and dried by using a GeneVac miVac sample concentrator (SP Scientific, Warminster, PA, USA). PDP and HD lipid extracts were reconstituted in 400 μl isopropanol:methanol:chloroform (4:2:1, v:v:v, containing 0.01% BHT) while SEC fraction lipid extracts were reconstituted in 200 μl isopropanol:methanol:chloroform (4:2:1, v:v:v, containing 0.01% BHT). All lipid extracts were stored in SureSTART™ glass screw top, level 3 high performance vials (Thermo Scientific).

### Direct infusion nano-ESI-ultrahigh resolution accurate MS lipidome analysis

Ten microliters of PDP lipid extract, 20 μl of HD lipid extract and 30 μl of EVs fraction lipid extracts were aliquoted to individual wells of a twin-tec® 96-well plate (Eppendorf, Hamburg, Germany), dried and then reconstituted in 40 μl (PDP and HD) and 15 μl (EVs fractions) of isopropanol:methanol:chloroform (4:2:1, v:v:v) containing 20 mM ammonium formate, respectively. The 96-well plate was then sealed with Teflon Ultra Thin Sealing Tape prior to MS analysis. Ten microliters of lipid sample was then aspirated and introduced via nano-ESI to an Orbitrap Fusion Lumos mass spectrometer (Thermo Fisher Scientific, San Jose, CA) using an Advion Triversa Nanomate (Advion, Ithaca, NY) operating with a spray voltage of 1.3 kV and a gas pressure of 0.3 psi in both positive and negative ionization modes. For MS analysis, the radio frequency lens was set at 10%. Full scan mass spectra were acquired at a mass resolving power of 500,000 (at 200 *m/z*) across a *m/z* range of 150–1600 using quadrupole isolation, with an automatic gain control (AGC) target of 2e5, and a normalized AGC target of 50%. The maximum injection time was set at 100 ms. Spectra were acquired and averaged for 1.5 min. Samples were measured in triplicates.

### Lipid identification, quantitation, and data analysis

“Sum composition” level lipid identifications were achieved using LipidSearch Software (ver 5.1.6, Mitsui Knowledge Industry, Tokyo, Japan) by automated peak peaking and searching against a user-defined custom database of lipid species (including the deuterated internal standard lipid species). The parent tolerance was set at 3.0 ppm, a parent ion intensity threshold three times that of the experimentally observed instrument noise intensity, and a max isotope number of 1 (i.e., matching based on the monoisotopic ion and the M+1 isotope), a correlation threshold (%) of 0.3 and an isotope threshold (%) of 0.1. The lipid nomenclature used here follows that defined by the LIPID MAPS consortium (28, 29). Information regarding lipid identification from positive and negative ionization modes is summarized in Supplemental Table S1. Due to potential isomeric overlap between even-numbered and odd-numbered acyl chain 1) phosphocholine (PC)+H^+^ ions and PE+H^+^ ions and 2) PS+H^+^ ions and PG+NH_4_^+^ ions in positive ionization mode, PC, PE, PS, and PG lipids were identified in negative ionization mode. Furthermore, due to the potential isomeric overlap between even-numbered acyl chain PC+HCOO^-^ and odd-numbered acyl chain PS-H^-^ ions in negative mode, only even-chain PC and PS lipid species were included in downstream analysis. Potential isomeric ether-containing lipids were annotated as O-/P- species. Lipid ions detected in at least 2 of 3 technical replicate measurements in any sample were included for quantitation and downstream analysis. Given the limited availability of internal standards across all lipid classes, only relative quantification was performed. Semiquantitative analysis of the abundances of identified lipid species was performed using an in-house R script, by comparing the identified lipid ion peak areas to the peak areas of the internal standard for each lipid class or subclass (if available, otherwise other structurally similar internal standards, see Supplemental Table S1). For statistical analysis, missing values were replaced with one-third of the minimum value of the entire dataset.

To account for natural differences in lipid composition and total lipid content between samples, internal standard-normalized abundances were further normalized to total identified lipid abundance prior to comparison. Then, the level of lipid enrichment or depletion at the lipid category and lipid class levels for EVs enriched fractions compared to PDP or HD were expressed as Log2 normalized abundance fold change (NAFC). Significant differences in Log2NAFC values were determined by one sample *t* test followed by multiple testing Benjamin–Hochberg false discovery rate (FDR) correction, with adjusted ∗*P* < 0.05, ∗∗*P* < 0.01, and ∗∗∗*P* < 0.001.

### Proteomics sample preparation

After lipid extraction, the remaining protein pellets were air-dried overnight. The PDP, LD, and HD pellets were dissolved in 200 μl 5% SDS in PBS solution and SEC fraction pellets were dissolved in 80 μl and heated at 95°C for 10 min with agitation. Protein content was again determined by Pierce™ BCA assay kit (Thermo Fisher Scientific) according to the manufacturer’s instructions. Protein reduction, alkylation, and digestion were carried out via suspension trapping using the micro S-Trap (ProtiFi) cartridges following the manufacturer’s instruction with slight modifications (30, 31, 32). Briefly, 3.5 μg protein from each sample was transferred to protein low bind tubes, reduced, and alkylated by 10 mM tris (2-carboxymethyl) phosphine and 40 mM chloroacetamide in 50 mM triethylammonium bicarbonate (TEAB) buffer at 99°C for 5 min. The reaction was then quenched in 1.2% phosphoric acid and a 7X volume of binding buffer (0.1 M TEAB and 90% methanol solution, pH 7.1) was added to each sample to generate the protein particles. Protein suspensions were transferred to micro S-Trap cartridges followed by centrifugation at 4,000 *g* for 1 min to remove solvent and trap protein particles. Protein bound to the quartz membrane was washed with 150 μl binding buffer three times and digested with Pierce™ Trypsin Protease MS-Grade (Thermo Fisher Scientific) dissolved in 50 mM TEAB buffer with digestion ratio of 1:20, trypsin:protein, w:w. Digestion occurred in a 37°C incubator for overnight. Digested peptides were eluted with three subsequent buffers, 40 μl of 50 mM TEAB buffer, 40 μl 0.2% formic acid, and 40 μl 50% aqueous acetonitrile (ACN) containing 0.2% formic acid. Protein solution was dried and reconstituted in 21.6 μl of 2% ACN containing 0.05% trifluoroacetic acid. PDP, HD, 6B-F10, 35nm-F10, 4B-F10, and 70nm-F11 protein suspensions were processed and analyzed in triplicate on different days to assess reproducibility of the proteomic workflow.

### DIA-LC-MS/MS proteomics analysis

For each sample, 0.7 μg of the peptide digests were introduced to an Acclaim Pepmap nano-trap column (Dionex-C18, 100 Å, 75 μm × 2 cm) using an isocratic flow rate of 5 μl/min in 2% ACN + 0.05% trifluoroacetic acid for 6 min. The trap column was then switched in-line with the Acclaim Pepmap RSLC analytical column (Dionex-C18, 100 Å, 75 μm × 50 cm) and peptides then analyzed using an Orbitrap Eclipse Tribrid mass spectrometer (Thermo Fisher Scientific). Solvent A comprised 0.1% formic acid (v/v) and 5% DMSO in water (v/v). Solvent B comprised ACN + 0.1% formic acid (v/v) and 5% DMSO in water (v/v). Each sample was eluted with the following gradient at a flow rate of 0.3 μl/min. The gradient started at 3%–23% solvent B over from 6 to 95 min, increased from 23% to 40% solvent B from 95 to 105 min, increased from 40% to 80% solvent B from 105 to 110 min, stabilized at 80% solvent B for 5 min, lowered to 3% solvent B over 0.1 min, and stabilized at 3% until finish at experimental run time of 125 min. Samples were delivered via nano-spray infusion with a spray voltage of 1.9 kV in positive ionization mode, with the ion transfer tube temperature set at 275°C. The full scan MS spectra data acquisition parameters included wide quad isolation; desired minimum points across the peak of 6; detector type, orbitrap mass resolution of 120,000; scan range of 350–1400 *m/z*; maximum injection time, 50 ms; normalized AGC target, 40%; microscans, 1; polarity, positive; and radio frequency lens of 30%. The default charge group state was set as 2. For higher-energy collisional dissociation MS/MS data acquisition, orbitrap with mass resolution was 30,000; higher-energy collisional dissociation collision energy was 32%, the desired minimum points across the peak was 9, collision energy type, normalized; isolation mode, quadrupole; multiplex ions disabled; window placement optimization disabled; number of scan events, loop count, 20; loop control, 3; loop time, 3; mass range, normal; precursor mass range, 361–1033; scan range, 200–2000 *m/z*; maximum injection time, 55 ms; normalized AGC target, 2000%; microscans, 1; data type, centroid; polarity, positive; and source fragmentation disabled. Precursor mass range of 360.5–1033.5 *m/z*, with an isolation window of 13.7 *m/z*, and an overlap window of 1 *m/z*. Supplemental Fig. S1 shows the reproducibility and robustness of the protein preparation and DIA proteomic analysis workflow of the SEC comparison.

### DIA proteomics data analysis and protein identification

MS raw files were analyzed using the Spectronaut v.14 (Biognosys, Switzerland) with library-free “direct-DIA” approach. The spectra fragment lists were searched against the SwissProt *Homo Sapiens* proteome FASTA library (downloaded on 20220829) for protein identification. The search parameters are as follows. Under Pulsar Search peptide setting, enzymes: trypsin/P, digest type: specific, max. peptide length: 52, min. peptide length: 7, missed cleavages was set as 2. Max variable modifications, 5, fixed modification: carbomidomethyl (C); variable modification: acetyl (protein N-term) and oxidation (M). Under DIA analysis setting, for identification, Q value cut-off was set as 1% for identification at precursor and protein levels. Single hit proteins were excluded, and proteins were defined by stripped sequence. FDRs for peptide spectrum match, peptide, and protein group were set to 0.01; quantification was performed with default settings: identified Q value for precursor filtering; imputation strategy and proteotypicity filter were none.

Protein label-free quantification (LFQ) method was set as automatic, quantity MS level was MS2 and quantity type was area. Cross-run normalization was enabled, and normalization strategy was set as automatic. Major protein grouping was performed by protein group ID; minor peptide grouping was performed by stripped sequence; major group quantity was based on mean peptide quantity; minor group quantity was based on mean precursor quantity. Default parameters were used for chromatograms extraction, calibration, workflow, and pipeline mode. After searching and filtering, enrichment analyses for gene ontology, Kyoto Encyclopedia of Genes and Genomes pathways, and Reactome pathway for EVs enriched fractions were performed using GeneCodis4 with default significance threshold.

## Results

EVs and lipoprotein particles share similar physical properties, such as size and density, often leading to coisolation (8, 9). Many studies on the protein and lipid content of plasma EVs have used suboptimal isolation methods that do not effectively remove non-EV particles. Similarly, suboptimal isolation methods or highly selective methodologies (i.e., antibody immunocapture) have been utilized for blood EV isolation, leaving the molecular compositions of global blood EVs unclear. Consequently, this has hindered further exploration of their functions or potential as biomarkers. To help address this issue, we adapted a previously published orthogonal method (22), which combines DGUC and SEC, which is essential for both enriching plasma EVs while simultaneously depleting lipoprotein particles and protein coisolates (Fig. 1). Four 4 different SEC columns were tested (qEVoriginal™ 35 nm and 70 nm, and home-made columns packed with either Sepharose™ CL-4B or CL-6B resin), to test their efficacy in depleting HDLs from EVs. Eluted fractions were analyzed by immunoblotting, proteomics, and lipidomics to validate the method and determine if depletion of plasma proteins and lipoproteins could enable detection of more EV-associated proteins.

### Validation of the DGUC and SEC EV isolation method, and SEC column comparison

**Fig. 2:**
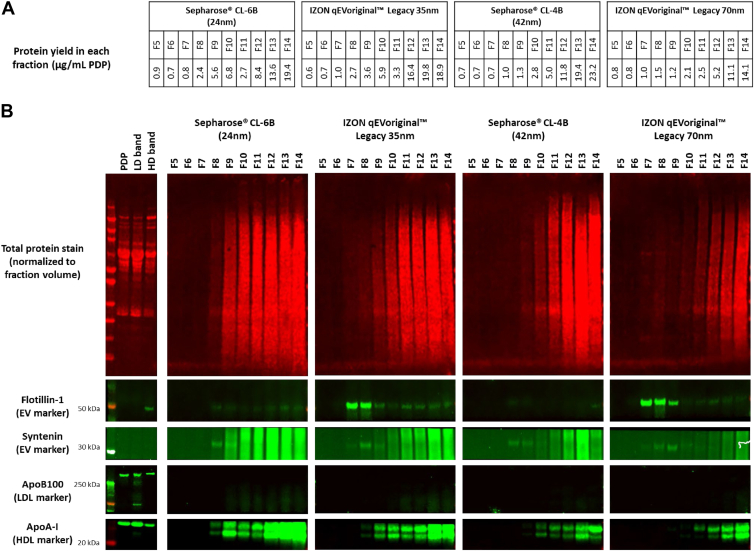
Protein quantitation and immunoblot characterization of plasma EVs isolated by density gradient ultracentrifugation (DGUC) and size-exclusion chromatography (SEC). A: Protein yield in each particle fraction from different SEC columns. B: Immunoblotting of PDP, LD, and HD samples (2.3 μg) and SEC fractions (equal volume). Samples were electrophoresed for 60 min at 160 V. Total proteins were stained and imaged, followed by immunoblotting with EV markers, flotillin-1 and syntenin, and *ApoB100* (LDL marker), and *ApoA-I* (HDL marker). EV = extracellular vesicle, PDP = platelet depleted plasma, LD = low density, HD = high density.

The protein yield in eluted SEC fractions is shown in Figure 2A and proteins visualized by total protein stain (Fig. 2B). Immunoblotting showed that syntenin, an endosome-derived EV marker, was enriched in F8 and F9 from Sepharose™ CL-6B, F7-9 from qEVoriginal™ 35 nm, F8 and F9 from Sepharose™ CL-4B, and F7-9 from qEVoriginal™ 70 nm, suggesting that these fractions contained EVs (Fig. 2B). The generic EV marker, flotillin-1, was detected in fractions eluted from the IZON SEC columns. Negligible levels of *ApoB100* were detected, indicating that LDLs (*ApoB100* rich) were reduced following DGUC. Compared to the qEVoriginal™ 70 nm, *ApoA-I* levels and total protein were higher in EV-containing fractions from all other SEC columns, likely due to their lower separation efficiency.

**Fig. 3:**
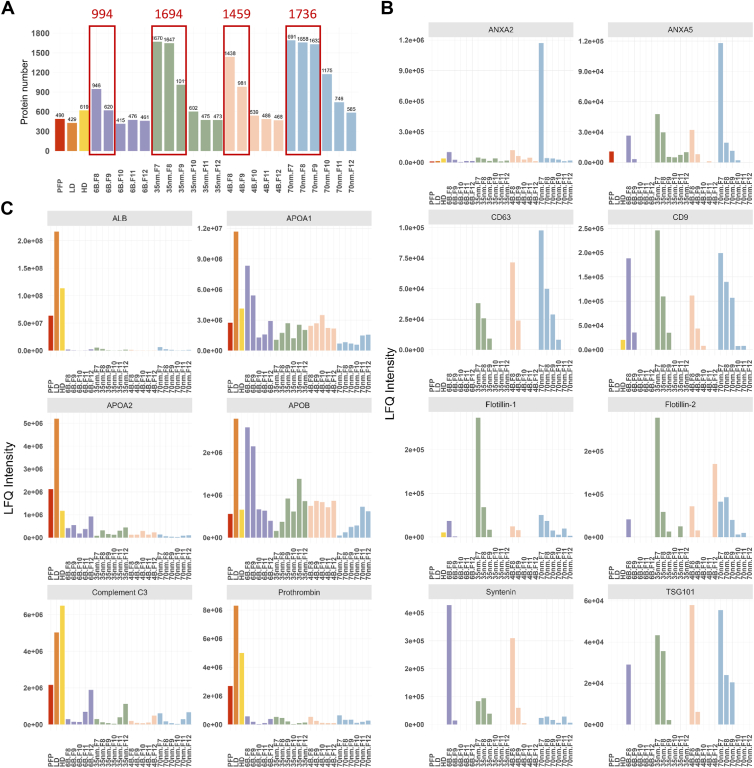
Size-exclusion chromatography (SEC) columns vary in their ability to enrich for EVs and deplete non-EV particles and abundant plasma proteins following density gradient ultracentrifugation (DGUC). A: Number of protein identifications in each sample. The EVs enriched fractions are highlighted by the red boxes and the numbers in red indicate the number of proteins identified in these fractions. B: The normalized LFQ intensity comparison of selected EV markers. The EVs enriched fractions contained higher level of EV markers (*ANXA2*, *ANXA5*, *CD63*, *CD9*, flotillin-1, flotillin-2, syntenin, and *TSG101*). C: The normalized LFQ intensity comparison of contaminating proteins. The EVs enriched fractions contained substantially low intensity of contaminant proteins (*ALB*, *APOA1*, *APOA2*, *APOB*, complement C3, and prothrombin). PDP = platelet depleted plasma, in red; LD band = low-density band, in orange; HD band = high-density band, in yellow; 6B = Sepharose™ CL-6B, in purple; 35 nm = qEVoriginal™ 35 nm, in green; 4B = Sepharose™ CL-4B, in pink; 70 nm = qEVoriginal™ 70 nm in blue, LFQ = label-free quantification.

To more thoroughly evaluate the benefit of removing abundant plasma proteins and lipoproteins from EV preparations, the PFP, LD, HD, and SEC fractions were submitted to proteomic analysis which identified 490, 429, and 619 proteins in PFP, LD, and HD, respectively. EVs isolated with Sepharose™ CL-6B, qEVoriginal™ 35 nm, CL-4B, and qEVoriginal™ 70 nm yielded 994, 1694, 1495, and 1736 proteins, respectively, with the qEVoriginal™ 70 nm detecting the most EV-associated proteins (Fig. 3A, Supplemental Table S2).

All EV enriched fractions contained some EV proteins (*ANXA2*, *ANXA5*, *CD63*, *CD9*, *FLOT1*, *FLOT2*, *SDCBP*, and *TSG101*), though their levels varied or where absent in some cases (Fig. 3B). The qEVoriginal™ 70 nm SEC column provided the most highly enriched EVs following DGUC, as evidenced by the relatively high LFQ intensity of known EV proteins (*ANXA2*, *ANXA5*, *CD63*, *CD9*, and *TSG101*) and relatively low LFQ intensity of known HDL proteins (*ALB*, *APOA1*, *APOA2*, *APOB*, complement C3, and prothrombin) compared to the EV fractions eluted from the other SEC columns (Fig. 3B–C). Analysis of the identified proteins of the EV fractions (F7–F9) using DGUC followed by qEVoriginal™ 70 nm SEC using gene ontology, Kyoto Encyclopedia of Genes and Genomes pathway, and Reactome enrichment analysis further validated the identity of EVs and confirmed the utility of this isolation approach (Supplemental Fig. S2). The same EV isolation strategy has previously been used by us to enrich EVs from plasma, with membrane enclosed vesicles confirmed by transmission electron microscopy (33).

**Fig. 4:**
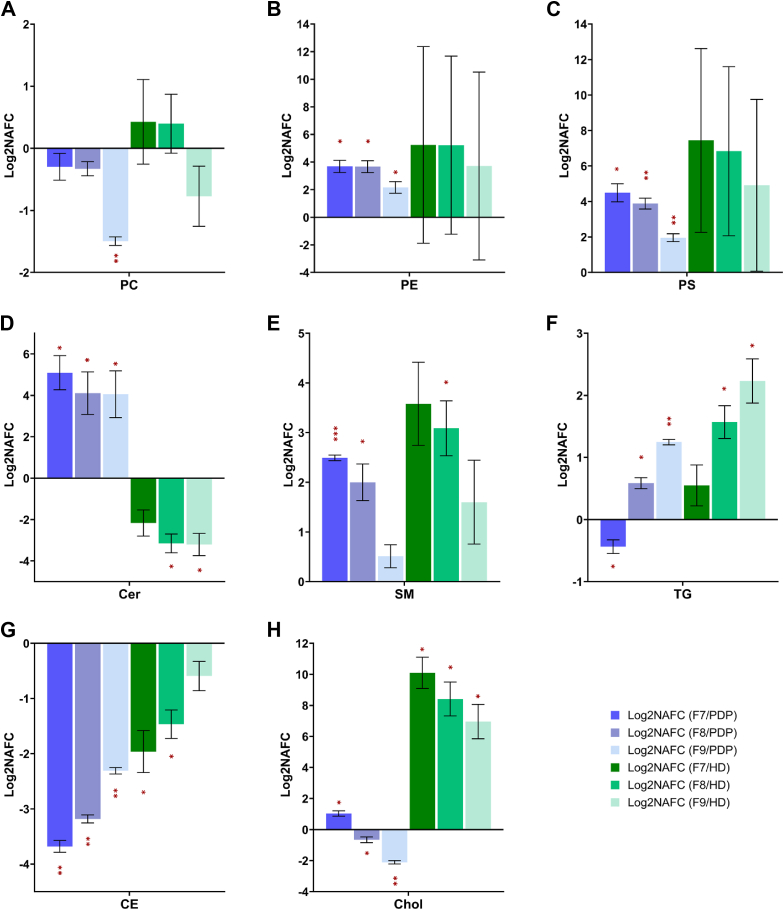
Comparison of lipid class in F7-9 EV fractions from qEVoriginal™ 70 nm SEC column against PDP and HD. Comparison is shown at (A) PC, (B) PE, (C) PS, (D) Cer, (E) SM, (F) TG, (G) CE and (H). Chol lipid class level. Data represent log2-transformed normalized abundance fold change (NAFC) ± standard deviation. One sample *t* test on the log2 NAFC values followed by multiple testing Benjamin–Hochberg FDR correction was used to determine statistical significance, with adjusted ∗*P* < 0.05; ∗∗*P* < 0.01, and ∗∗∗*P* < 0.001. PDP = platelet depleted plasma, EV = extracellular vesicle; PC = phosphocholine, PE = glycerophosphoethanoamine, PS = glycerophosphoserine, Cer = ceramide, SM = sphingomyelin, TG = triglyceride, CE = cholesteryl ester, and Chol = cholesterol.

To further evaluate and validate the DGUC and SEC isolation method, we then performed lipidomic analysis to investigate the level of lipid enrichment and depletion in the EVs compared to PDP and HD. Lipids were identified across four main lipid categories, including glycerophospholipids (GP), SP, glycerolipids, and sterol (ST) lipids (Supplemental Tables S3 and S4). Figure 4 presents the Log2NAFC of lipid classes in the F7–F9 fractions compared to PDP and HD. Depletion of glycerophosphocholine (PC) was observed in F7–F9, but most significantly in F9, compared to PDP (Fig. 4A). The EV fractions were significantly enriched in glycerophosphoethanoamine (PE), glycerophosphoserine (PS), ceramide (Cer) and sphingomyelin (SM) lipids when compared to PDP (Fig. 4B–E). When compared to the HD fraction, fractions F8 and F9 were depleted in Cer (Fig. 4D). Triglyceride (TG) lipids were significantly depleted in F7 but higher in the F8 and F9 than PDP (Fig. 4F), suggesting potential “contamination” with non-EV components. CE lipids, major lipid components in lipoprotein particles, were consistently depleted in all EV fractions in comparison to PDP and HD (Fig. 4G), suggesting effective depletion of lipoprotein particles in the EV preparation. Interestingly, the F7 fraction, but not F8 or F9, showed enrichment of free cholesterol (Chol) compared to PDP (Fig. 4H), potentially suggesting the presence of distinct EV subtypes in each fraction.

### Proteomic characterization of EVs isolated from healthy volunteers

**Fig. 5:**
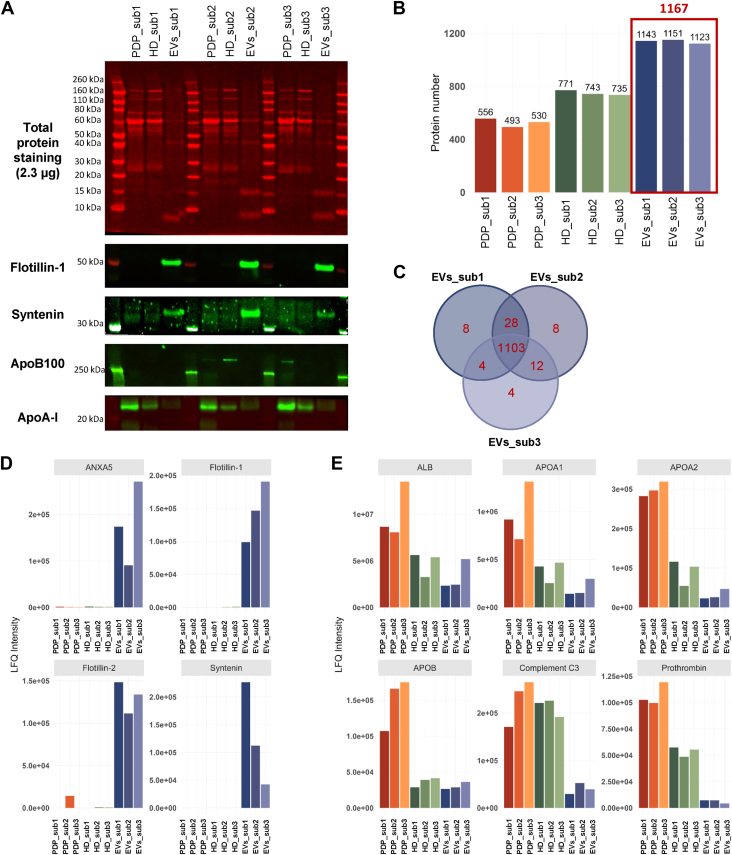
Immunoblot and proteomic characterization of highly enriched plasma EVs. A: Normalized PDP, HD and EVs samples (2.3 μg) were electrophoresed for 60 min at 160 V. Total proteins were stained, followed by immunoblotting with *ApoB100* (LDL marker), *ApoA-I* (HDL marker), and syntenin and flotillin-1 (general EVs markers). B: The number of proteins identified in each sample (PDP, HD, and EVs). The red frame and number indicate total proteins identified. C: Venn diagram of identified proteins in plasma EVs. D: The normalized LFQ intensity comparison of representative EVs markers. E: The normalized LFQ intensity comparison of plasma proteins and lipoproteins. PDP = platelet depleted plasma, HD band = high-density band, EV = extracellular vesicle, sub = volunteer subject, LFQ = label-free quantification.

With confidence in our plasma EV enrichment method, we next assessed its reproducibility and robustness using samples from three healthy individuals. Plasma EVs were isolated from three young healthy female volunteers. On average, 1 ml of PDP yielded EVs equivalent to 3.71 ± 0.28 μg protein. Immunoblotting analysis showed that EVs from the three individuals were enriched in flotillin-1 and syntenin, had limited ApoB100 and contained negligible ApoA-I protein (Fig. 5A). Proteomic analysis of the combined EV fractions resulted in the identification of 1143, 1151, and 1123 proteins (Fig. 5B) with the protein content similar between individuals (Fig. 5C), with enrichment in EV proteins and depletion of blood contaminants (Fig. 5D, E) relative to that from PDP.

**Fig. 6:**
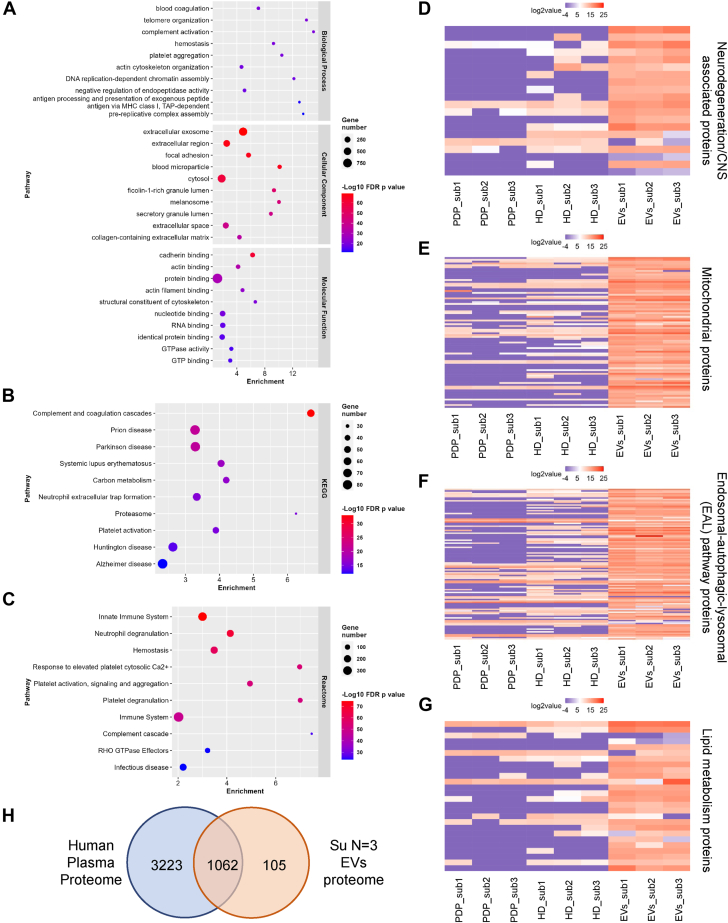
Enrichment analysis of the proteome of highly enriched plasma EVs. A: Gene ontology enrichment analyses including top 10 biological process, cellular component, and molecular function pathways. B: Top 10 enriched KEGG pathways. C: Top 10 enriched Reactome pathways. D: Venn diagram showing numbers of proteins identified in plasma EVs compared with Human Plasma Proteome (Deutsch *et al.*, 2021). Histograms show identification and enrichment of (E) neurodegeneration/CNS associated proteins, (F) mitochondrial proteins, (G) endosomal-autophagic-lysosomal (EAL) pathway proteins, and (H) lipid metabolism proteins in EVs. The databases were from Human Protein Atlas databases and KEGG pathways. PDP = platelet depleted plasma, HD band = high-density band, EV = extracellular vesicle, sub = volunteer subject, CNS = central nervous system, KEGG = Kyoto Encyclopedia of Genes and Genomes.

Gene ontology enrichment analysis of EVs further validated the identity of the EVs (Fig. 6A), and highlighted their proteomes associated with CNS disease pathways (Fig. 6B), the immune system, and platelet activity (Fig. 6C). We identified neurodegeneration/CNS-associated proteins (Fig. 6D) such as *MBP*, *SYN1*, *SYT1*, *NRGN*, *TMEM63A*, *SNCA*, *SOD1*, and *NCSTN* (full list in Supplemental Table S5) in EVs. Moreover, proteins associated with mitochondria (82 proteins), and the EAL pathways (107 proteins) in EVs (Fig. 6E, fig6F, respectively), highlight the interplay of these intracellular pathways with EV biogenesis. Twenty-six lipid metabolism proteins were also identified in EVs (Fig. 6G), indicating a potential role for EVs in regulating lipid homeostasis in recipient cells. Notably, these proteins were either undetectable or present at substantially low levels in PDP. The EV proteins associated with these pathways are provided in Supplemental Table S5. When comparing the EV proteome with the Human Plasma Proteome (34), 105 proteins were detected in our enriched EVs that have not been reported in the Human Plasma Proteome (Fig. 6H and Supplemental Table S6). These were predominantly CNS, mitochondrial, histone, and EAL-associated proteins. Supplemental Fig. S3 and Supplemental Table S7 show the relative enrichment of CNS, mitochondrial, EAL, and lipid metabolism-associated proteins in the EVs enriched fractions isolated using different SEC columns following DGUC. The qEVoriginal™ 70 nm SEC column isolated EVs gave rise to the most lipid metabolism proteins (65 proteins, highlighted in Supplemental Table S8 with the corresponding lipid metabolism pathways), suggesting their potential role in regulating lipid metabolism in recipient cells. Notably, several SP-regulating enzymes (*SPTLC2*, *ASAH1*, *GLB1*, *NEU2*, and *ARSA*) were identified (Supplemental Table S8). *SPTLC2* initiates SP biosynthesis, *ASAH1* breaks down Cer, and *GLB1*, *NEU2*, and *ARSA* are involved in glycosphingolipid degradation. The presence of these enzymes may suggest active involvement in SP metabolic processes, consistent with the enrichment of SP content, particularly Cer and SM, in the EVs (Figure 4D, E). The proteomic data show that, although only three individuals were assessed, consistent plasma EV profiles were observed, including for low-abundant proteins. This consistency highlights the utility of the reported method for reducing contaminants and, consequently, sample complexity.

### Lipid enrichment and depletion in plasma EVs isolated from healthy volunteers

Numerous studies have attempted to analyze either the proteome or lipidome of EVs from plasma and serum, but limitations in isolation methods have constrained their findings (13, 14, 15, 16, 17, 18, 19, 20, 21). While three previously reported studies have used orthogonal techniques to isolate plasma EVs, they used less advanced MS technologies to those employed here, limiting the depth of the results obtained, while none have reported enrichment or depletion of EV lipids compared to PFP or HD (22, 23, 24).

**Fig. 7:**
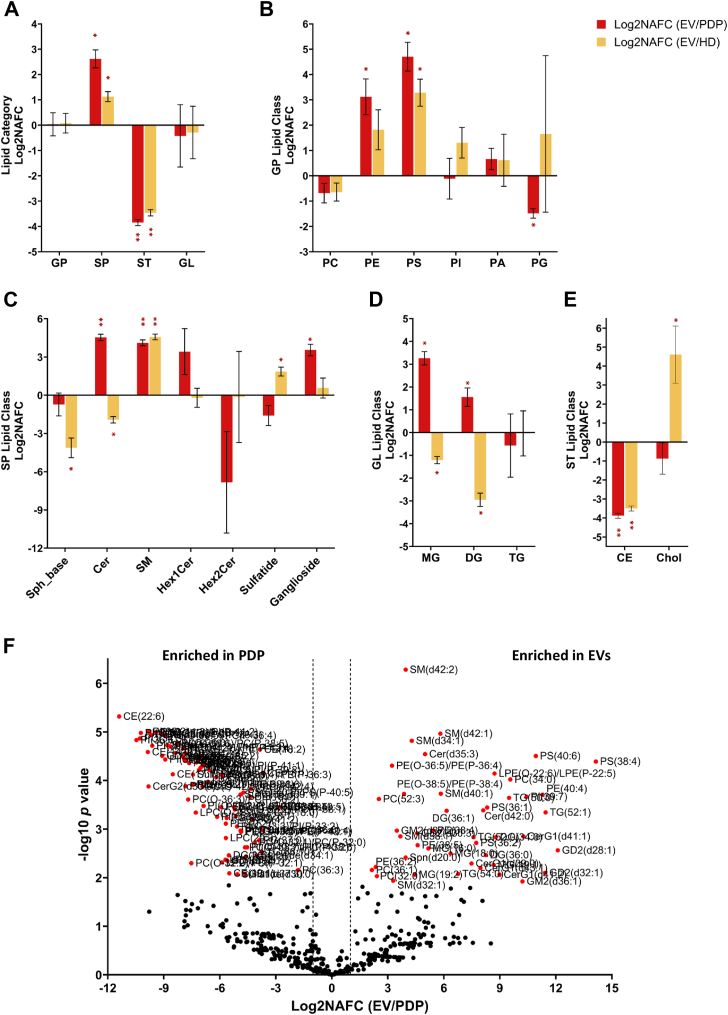
Comparison of lipid in EVs against PDP and HD. A: Comparison at lipid category level. Four lipid categories include glycerophospholipids (GPs), sphingolipids (SPs), glycerolipids (GLs), and sterol (ST) lipids. Comparison at (B) GP lipid class level, (C) SP lipid class level, (D) GL lipid class level and (E.) ST lipid class level. Data represent log2-transformed normalized abundance fold change (NAFC) ± standard deviation. One sample *t* test on the log2 NAFC values followed by multiple testing Benjamin–Hochberg FDR correction was used to determine statistical significance, with adjusted ∗*P* < 0.05; ∗∗*P* < 0.01. F: Volcano plot showing comparison between PDP and EVs at lipid sum composition level. Multiple *t* test followed by Benjamin–Hochberg FDR correction was used to determine statistical significance. Significantly differential lipids with adjusted *P* value < 0.05 are highlighted in red. PDP = platelet depleted plasma, HD band = high-density band, EV = extracellular vesicle, PC = phosphocholine, PE = glycerophosphoethanoamine, PS = glycerophosphoserine, PI = glycerophosphoinositol, PA = glycerophosphoric acid, PG = glycerophosphoglycerol, Sph_base = sphingoid base, Cer = ceramide, SM = sphingomyelin, Hex1Cer = hexosyl-1-ceramide, Hex2Cer = hexosyl-2-ceramide, MG = monoglyceride, DG = diglyceride, TG = triglyceride, CE = cholesteryl ester, Chol = cholesterol, FDR = false discovery rate.

Here, we identified lipids from the combined plasma EV containing fractions from three individuals and compared them with the PDP and HD to determine the lipids that are enriched and depleted during EV isolation (Fig. 7). A total of 526 lipid species were identified at the sum composition level (summarized in Supplemental Table S9 and S10). At the lipid category level, EVs were significantly enriched in SP lipids and depleted in ST lipids compared to PDP and HD (Fig 7A). For the GP lipid category, EVs exhibited a significant increase in log2NAFC PE and PS lipids and depletion in PG lipids in comparison to PDP (Fig. 7B). The observed enrichment in SP lipids in the EVs was primarily due to a significant increase in Cer and SM lipids (Fig. 7C). Interestingly, MG and DG lipids were found to be enriched, albeit with no significant difference observed between EVs and PDP or HD (Fig. 7D). Given these are the combined EVs fractions, as illustrated above, there are likely a heterogenous pool of EV populations. For the ST lipid category, CE lipids were depleted in EVs when compared to PDP or HD (Fig. 7E), while Chol was significantly enriched in EVs compared to HD. The observed decrease in total CE lipids and the increase in SP lipids in EVs compared to PDP was further observed through the depletion of individual CE lipids, CE(18:1), CE(18:2), CE(22:6), CE(14:0), and the enrichment of individual SP lipids, SM(d42:2), SM(d42:1), SM(d34:1), SM(d40:1), SM(d32:1), Cer(d42:0), and Cer(d35:3), as shown in Fig. 4F. Other lipids enriched in EVs included PS(38:4), PS(40:6), PS(36:1), PE(36:2), PE(38:5), PE(40:4), and several ether-containing PE species, and gangliosides, which have been linked to neurodegenerative diseases, inflammatory disorders, and cardiovascular conditions (27, 35, 36, 37). Improved detection of these lipid species through the isolation of highly enriched EVs could enhance disease diagnosis and provide deeper insights into lipid-related pathologies.

## Discussion

The composition of EVs in plasma has broad implications for understanding their associated biological functions, in disease diagnosis and therapeutic potential. However, the complexity of plasma poses challenges in determining the molecular compositions of EVs amongst the various other cell-free components that often coisolate. This study employed a nonselective isolation strategy, combining DGUC and SEC methodologies, with a focus on capturing a representative, albeit likely heterogeneous pool of EVs that are significantly depleted in “contaminating” high abundance plasma proteins and lipoprotein particles. We then applied this approach reveal proteins in EVs associated with mitochondria, EAL pathways, and the CNS, demonstrating the benefits of reducing sample complexity to enable the detection of these proteins relative to other studies. Although absolute EV lipid quantification was not feasible in this study due to varying lipid-protein ratios and lipid compositions across samples, and compared to PFP and HD, identified lipid abundances normalized to internal standards to account for technical variability, and then further normalized to total lipid abundance for comparison, enabled an assessment of PE, PS, Cer, and SM lipid enrichment relative to PDP and HD in EVs, and depletion of CE relative to PDP. Reporting CE lipid abundance in blood EV preparations is crucial because CE lipids are major components of lipoprotein particles and is recommended to be used to assess the efficacy of future methods developed for EV isolation and removal of lipoproteins from plasma EV preparations (38).

Some studies have previously reported the proteome (14, 16, 18, 20, 22, 24, 39, 40, 41, 42) and lipidome of EVs in blood (17, 19, 21). However, the large majority of isolation methods used in these studies are known to coisolate abundant plasma proteins and/or lipoproteins with EVs, thereby limiting their utility (43, 44). For molecular characterization therefore, albeit likely not suitable for use in high-throughput or clinical applications, combining DGUC with SEC should be considered one of the most effective methods for obtaining highly enriched EVs (18, 22, 23).

We evaluated the isolation of plasma EVs by monitoring the depletion of plasma proteins such as albumin and apolipoproteins. Our plasma EVs preparations had minute abundances of albumin (0.99% total protein abundance) and apolipoprotein (ApoA-I, ApoA-II and ApoB100 combined, approximately 0.08% total protein abundance). These values are significantly lower than what was reported in a recent meta-analysis investigating the protein profile of plasma EVs (note that our study used DIA where the meta-analysis was from data acquired using data-dependent acquisition MS methods), where the albumin level ranged from approximately 10–40% of the total protein abundance in the eight studies included in the meta-analysis (43).

We demonstrate that highly enriched plasma EVs may provide insights into the intracellular pathway associated with mitochondria, endosome, autophagosome, and lysosome, which have been implicated in diseases (45, 46). Eighty-two mitochondria proteins and 107 EAL pathway–associated proteins were detected in plasma EVs, including several that have not previously been detected in plasma. This is presumably due to the crosstalk between EVs biogenesis and mitochondrial and EAL pathways, which contribute to the cargo loaded into EVs (1, 2, 47, 48). In addition, CNS and neurodegeneration-associated proteins (*MBP*, *SYN1*, *SYT1*, *NRGN*, *TMEM63A*, *SNCA*, *SOD1* and *NCSTN* for example) were detected in the plasma EVs and were either not observed or present in minimal abundance in PDP. By depleting, contaminating blood proteins and lipoprotein particles from EVs of the ability to detect low-abundant proteins through MS-based proteomics was significantly enhanced. These low-abundant proteins could therefore now be detected in the periphery and potentially contribute to development of disease diagnostics and monitor strategies. A caveat to our study is the possibility that plasma was not completely platelet free. The minimal presence of platelets, however, does not undermine our findings as the results demonstrate the enrichment of proteins associated with specific cellular pathways and tissues in EVs compared to PDP.

As a fundamental component of EVs, lipids have gained increasing interest in terms of understanding their structural and biological roles, as well as their biomarker and therapeutic potential. Previous studies reporting the lipid content of plasma or sera EV preparations have commonly employed non-ideal isolation methodologies which do not deplete major blood non-EV lipids. The presence of these non-EV lipids can overshadow the bona fide lipids that constitute EVs, posing a challenge for the detection of low-abundant EV lipids with biomarker potential through MS. In previously published blood EV lipid studies, CE lipids have either not been identified, or direct evidence of CE lipid depletion in EVs compared to PDP or HDL has been limited (19, 21). Considering that lipoproteins comprise an inner core of CE, the depletion of CE lipid content in blood EVs preparations relative to PDP and HDL can serve as an indicator of lipoprotein depletion, and therefore enrichment of EVs. In this study, we provide a comparative lipidomic analysis between EV and their source material PDP and contaminating HD particles, confirming the relative depletion of CE in plasma EVs. Given the challenges in absolute quantitation between plasma, HD, and EVs, in the revised manuscript, we report NAFC of lipid enrichment or depletion in EVs, rather than absolute concentrations. In line with the recent review by Skotland *et al.* (38), we encourage future studies involving blood EV lipidomics to report the CE content of their EV preparations relative to PDP or HD, particularly when aiming to assess the efficacy of their isolation methodology in removing lipoproteins. Chol has been reported to make up close to 40% of total lipids in EVs, primarily evidenced in in vitro studies (49). To our knowledge, however, the proportion of free Chol within total blood EVs is unknown. Here, we did not observe enrichment of Chol in the EV fractions relative to PDP, while significant enrichment was observed was observed in EV compared to HD. This could be attributed to the significant abundance of Chol already present in plasma (reported as 10–20% total plasma lipid by (50, 51). In future studies, Chol concentrations (both absolute and relative) should be measured more accurately using corresponding internal standards. Lipoprotein particles have been reported to interact with and form a corona on the surface of EVs (52, 53). However, the results reported here suggest that the majority of lipoproteins in plasma are effectively depleted using the isolation methodology applied in this study.

EVs isolated from sources other than blood, such as cell lines, urine and adipose tissue, are known to be rich in SM, PS, and PE lipids (1, 26, 54, 55, 56, 57, 58, 59, 60). The results obtained in the current study indicate that plasma EVs are enriched in PE, PS, Cer, and SM lipids, compared to both the original source (PDP), and HD. The enrichment in SPs was supported by the presence of SP regulating enzymes that were identified in the EV enriched fractions, suggestive of the active involvement of lipid metabolism within the EVs. Peterka *et al.* used multiple mass spectrometric techniques to examine plasma EVs isolated by polymer precipitation (19). They observed no difference in the SM lipid composition between plasma and EVs and found that TG lipids comprised nearly 50% of total lipids in EVs, higher than that in plasma (30% of total lipids). However, the large TG content suggests contamination typically associated with polymer precipitation EV isolation (19). Sun *et al.* have also previously described the analysis of lipids from blood EVs and reported that PC are the dominant lipids (nearly 60 mol% total lipids), followed by SM (over 20 mol% total lipids) but did not report the presence of CE lipids in the isolated EVs. This discrepancy is also likely attributed to potential coisolation of other plasma components (21). SM, PE, PS, and Cer lipids have been flagged as potential biomarkers for numerous diseases, particularly in CNS disorders (35, 61, 62). For example, SM and Cer lipids can be early predictors of cognitive decline (63, 64) and Cer lipids related to apoptosis have implications in Lewy body disorders (65) and cardiovascular disease (36). Enriching these lipids from plasma, by isolating highly enriched EVs, enhances their signal to noise ratio, and could improve detection of these potential biomarkers in clinical applications. The further development of technologies for effective EV isolation, however, that are compatible with high-throughput processing, will be required for this to become a reality.

While the method we have reported here does not specifically separate the enriched EVs by cell type of origin, or subpopulation, our data do provide a glimpse into some of the subtypes of EVs in the population. It is evident that the different SEC columns provide varying capabilities in enriching EVs via the depletion of plasma and lipoprotein particle proteins, resulting in varying total protein numbers in the EV enriched fractions from the various SEC columns that were evaluated. The selection of different pore size SEC columns may contribute to the isolation of EV subpopulations, as seen by the differences in known EV marker intensities. Among the SEC columns examined, the qEVoriginal™ 70 nm SEC column has the largest pore size, allowing for more efficient separation of EVs from smaller HDL particles and blood protein. The three EV enriched fractions obtained from this column also likely contain different EV subtypes, as evidences by variations in their individual lipid and protein content. For example, the increased TG content observed in F8 and F9 EV fractions could indicate contamination from non-EV lipids, or the presence of a “lipoprotein corona” bound to the surface of those EV subtypes (52, 66). While other studies have examined and compared different SEC columns with various resins containing different pore sizes for separating EVs from lipoprotein particles and blood proteins using neat plasma sample (67, 68), our study used the HD fraction obtained by initial fractionation of the plasma using DGUC for SEC separation. Our reported evaluation and validation of the SEC columns’ efficacy in separating EVs and HDLs within the HD fraction will be valuable for researchers in selecting appropriate SEC columns for future EV preparation.

This study employed lipidomic and proteomic tools to evaluate the enrichment of EVs from plasma, depleted of lipoprotein particles and blood proteins, and provided direct evidence of lipid enrichment/depletion in EVs compared to PDP and HD. Albeit obtained with a small sample size, this study underscores the value of enriching EVs for improved MS analysis, to enhance the detection of EV associated proteins (including low-abundant proteins not otherwise observed in plasma), and lipids, which are highly conserved across individual plasma samples. Importantly, the potential reported here for EVs highly enriched from plasma to provide insights into intracellular pathways highlights the need for the continued development of techniques for enriching EVs from blood that are compatible for applications in translation and clinical settings for disease diagnosis and monitoring.

## Data availability

The datasets generated during and/or analyzed during the current study are available in ProteomeXchange.

## Supplemental data

This article contains supplemental data.

### Ethics approval

This study was performed in accordance with the National Health and Medical Research Council guidelines. Approval was granted by the Ethics Committee of Melbourne University Application Number 24582.

### Consent to participate

Informed consent was obtained from all individual participants included in the study.

## Conflict of interest

The authors declare that they have no conflicts of interest with the contents of this article.

## Supplementary data

Supplementary Tables

Supplementary Figures Supplementary Figure 1. Reproducibility of S-Trap proteomics sample preparation and DIA-proteomic analysis method. The samples, PDP, HD, 6B_F10, 35nm_F10, 4B_F10 and 70nm_F11 were processed and analyzed in triplicates. A. Boxplot shows the Log2 MS2 quantity distribution of the measured samples. B. The number of protein identifications from measured samples in triplicate. DIA = data independent acquisition, PDP = platelet depleted plasma, HD = high density band, 6B = Sepharose™ CL-6B, 35 nm = qEVoriginal™ 35 nm, 4B = Sepharose™ CL-4B, 70 nm = qEVoriginal™ 70 nm. Supplementary Figure 2. Enrichment analysis of the proteome of plasma EVs. A. Gene ontology enrichment analyses including Top 10 biological process, cellular component and molecular function pathways. B. Top 10 enriched KEGG pathways. C. Top 10 enriched Reactome pathways. The EVs were isolated from plasma using density gradient ultracentrifugation (DGUC) and qEVoriginal™ 70nm SEC (Fractions F7-9). EVs = extracellular vesicles. Supplementary Figure 3. Identification and relative enrichment of (A) neurodegeneration/CNS, (B) mitochondria associated proteins, (C) endosomal-autophagic-lysosomal (EAL) pathway, and (D) lipid metabolism proteins in PDP, LD, HD and particle fractions collected from density gradient ultracentrifugation (DGUC) followed by size exclusion chromatography (SEC). Low abundant EV-associated proteins are minimally detectable via MS of PDP, LD or HD were identified, and some were found enriched in EVs enriched fractions, specifically EVs isolated using the qEVoriginal™ 35 nm and 70 nm SEC columns. The databases were from Human Protein Atlas databases and KEGG pathways. PDP = platelet depleted plasma, LD= low density band, HD = high density band, 6B = Sepharose™ CL-6B, 35 nm = qEVoriginal™ 35 nm, 4B = Sepharose™ CL-4B, 70 nm = qEVoriginal™ 70 nm.

MIBlood_EV Standardized Reporting Tool
