# Supplementary figures and images for: Multiomics analysis to evaluate the enrichment of extracellular vesicles from human plasma
Source: J Lipid Res. 2025 Aug 19;66(9):100877. doi: 10.1016/j.jlr.2025.100877 (PMC12465043; doi:10.1016/j.jlr.2025.100877)

A

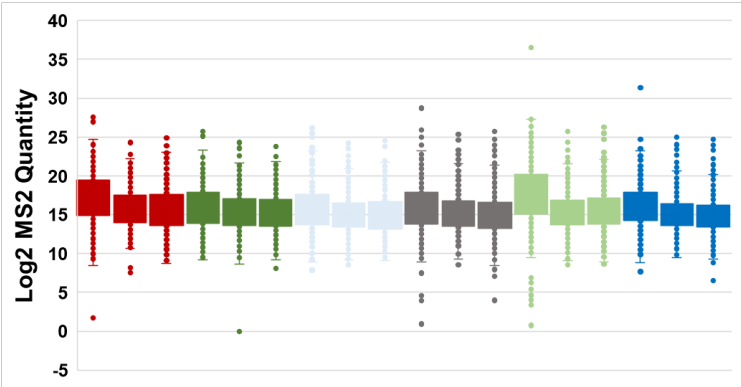

B

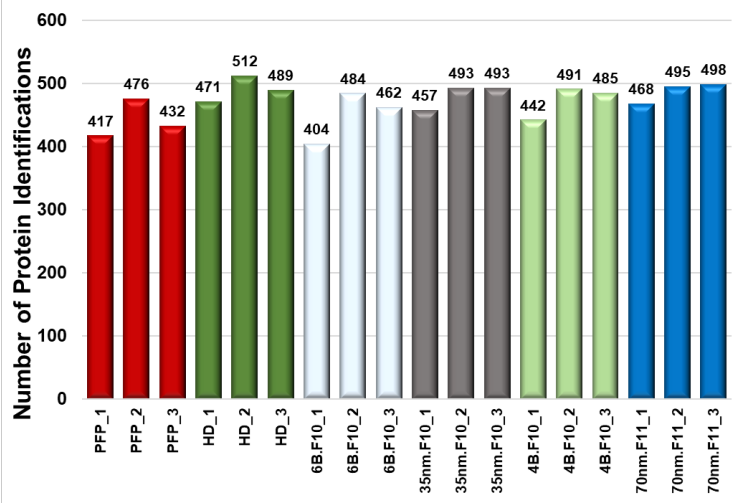

A

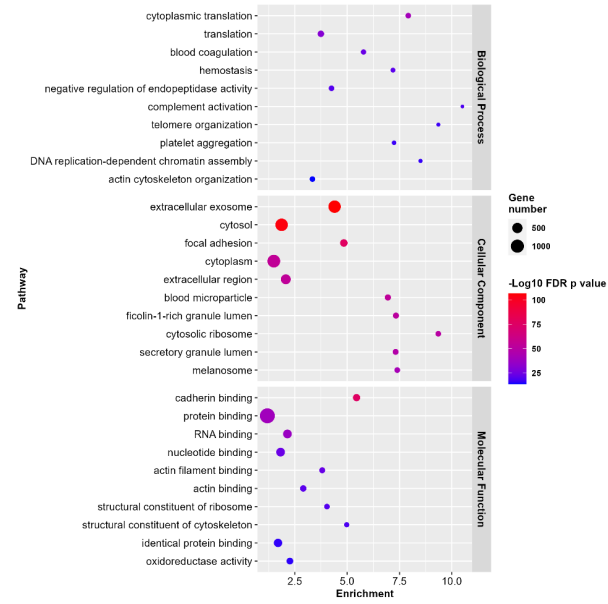

B

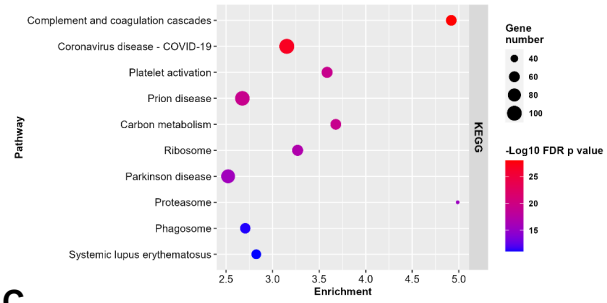

C

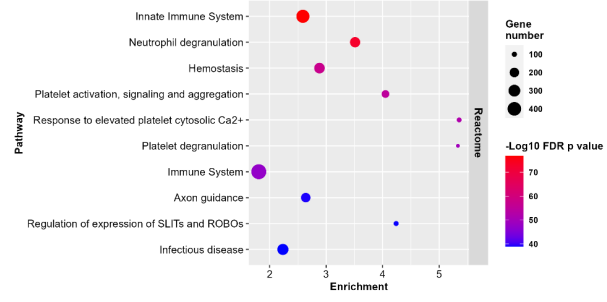

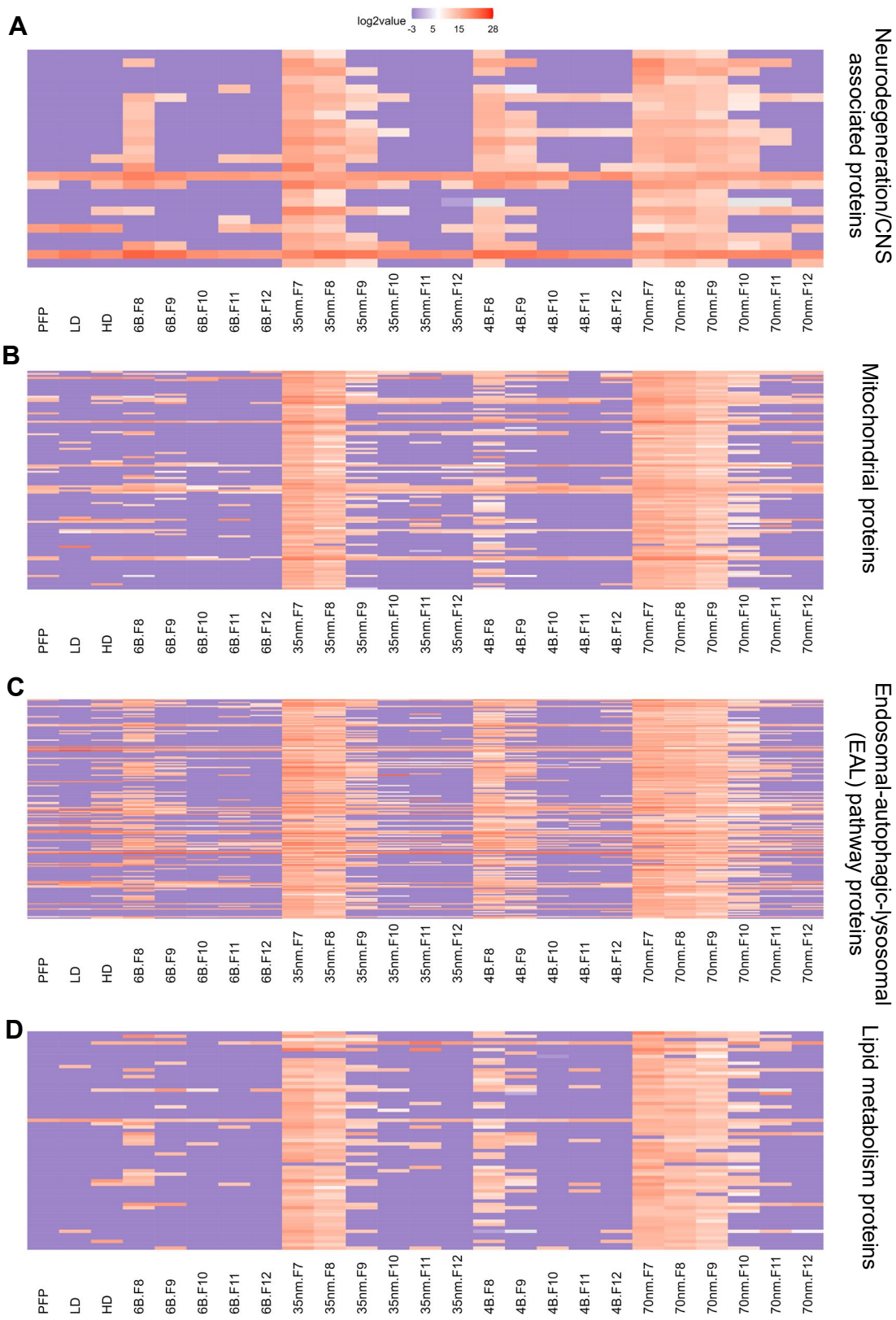

Supplement: Supplementary Figures — Supplementary Figure 1. Reproducibility of S-Trap proteomics sample preparation and DIA-proteomic analysis method. The samples, PDP, HD, 6B_F10, 35nm_F10, 4B_F10 and 70nm_F11 were processed and analyzed in triplicates. A. Boxplot shows the Log2 MS2 quantity distribution of the measured samples. B. The number of protein identifications from measured samples in triplicate. DIA = data independent acquisition, PDP = platelet depleted plasma, HD = high density band, 6B = Sepharose™ CL-6B, 35 nm = qEVoriginal™ 35 nm, 4B = Sepharose™ CL-4B, 70 nm = qEVoriginal™ 70 nm. Supplementary Figure 2. Enrichment analysis of the proteome of plasma EVs. A. Gene ontology enrichment analyses including Top 10 biological process, cellular component and molecular function pathways. B. Top 10 enriched KEGG pathways. C. Top 10 enriched Reactome pathways. The EVs were isolated from plasma using density gradient ultracentrifugation (DGUC) and qEVoriginal™ 70nm SEC (Fractions F7-9). EVs = extracellular vesicles. Supplementary Figure 3. Identification and relative enrichment of (A) neurodegeneration/CNS, (B) mitochondria associated proteins, (C) endosomal-autophagic-lysosomal (EAL) pathway, and (D) lipid metabolism proteins in PDP, LD, HD and particle fractions collected from density gradient ultracentrifugation (DGUC) followed by size exclusion chromatography (SEC). Low abundant EV-associated proteins are minimally detectable via MS of PDP, LD or HD were identified, and some were found enriched in EVs enriched fractions, specifically EVs isolated using the qEVoriginal™ 35 nm and 70 nm SEC columns. The databases were from Human Protein Atlas databases and KEGG pathways. PDP = platelet depleted plasma, LD= low density band, HD = high density band, 6B = Sepharose™ CL-6B, 35 nm = qEVoriginal™ 35 nm, 4B = Sepharose™ CL-4B, 70 nm = qEVoriginal™ 70 nm. [file mmc2.pdf]
